# Ileovesicostomy Update: Changes for the 21st Century

**DOI:** 10.1155/2009/801038

**Published:** 2009-10-27

**Authors:** W. Britt Zimmerman, Richard A. Santucci

**Affiliations:** Department of Urology, Detroit Receiving Hospital, Detroit Medical Center, Harper Professional Building, Suite 1017, 4160 John R., Detroit, MI 48201, USA

## Abstract

*Objectives*. To review the literature regarding ileovesicostomy and evaluate our patient population for clinical characteristics. *Methods*. Various surgical reconstructive techniques allow management of difficult clinical scenarios involving patients with neurogenic bladder, irretraceable lower urinary tract symptoms, lower urinary tract disaster, and urethrocutaneous fistulae. One such reconstructive technique employed is the ileovesicostomy. This procedure provides patients with a low-pressure urinary conduit utilizing the ileum and native bladder that empties without catheterization. We describe our patient population who underwent ileovesicostomy for 5 consecutive years ending 2007 at Detroit Receiving Hospital. *Results*. Most common diagnosis was neurogenic bladder secondary to spinal cord injury. Our population and clinical outcomes are similar to those previously reported in the literature. *Conclusions*. Based on our experience, we suggest that patients with severe lower urinary tract symptoms and who are unable to perform clean intermittent catheterization and/or refractory to medical therapy ileovesicostomy should be the procedure of choice.

## 1. Introduction

In the 21st century, various surgical reconstructive techniques allow management of difficult clinical scenarios involving neurogenic bladder, intractable lower urinary tract obstruction, and lower urinary tract disasters such as sometimes occur after prostate cancer treatments. As a method in dealing with the worst of these problems, suprapubic diversion has progressed significantly over the past 50 years. In the 1950s, cutaneous vesicostomy evolved into ileocystostomy (which is now called ileovesicostomy), which improved the location and quality of the stoma, allowing improved patients dryness [1, 2]. During the late 1900s, cystectomy and ileal loop also became common, but likely is an unnecessarily invasive treatment for many patients nowadays. From the 1960s to 1980s, ileal loop diversion and chronic indwelling urethral catheterization were the mainstays of therapy. In the 1970s, clean intermittent self-catheterization (CIC) [3] took the forefront, but more complex techniques such as ileovesicostomy are still commonly necessary in special or refractory cases.

## 2. History

Smith and Hinman first described ileovesicostomy in 1955, using dogs and anastomosing ileum to the native bladder in situ. This allowed the bladder to act as a “continent reservoir,” which was drained volitionally through the ileal conduit instead of the urethra. They postulated that the bladder neck continence mechanism would remain, and that voiding would occur through the subject's own detrusor contraction [1]. Of course, in humans, ileovesicostomy is designed as a completely incontinent suprapubic diversion.

In 1957, Cordonnier described a case series of three successive patients on whom he utilized an “ileocystostomy” (aka “ileovesicostomy”) for neurogenic bladder in children suffering from meningomyelocele. In Cordonnier's modification, the ileum was anastomosed to the bladder in a peristaltic fashion, and the urine was collected in a rudimentary urostomy bag [2].

In more recent years, multiple authors have reported that the ileovesicostomy procedure provides an easily emptying and noncatheterizable low-pressure urinary conduit [4–7]. With ileovesicostomy, the primary objectives include establishing a vesical diversion that has a low detrusor leak point pressure (as low as 8 cm in some series) and minimal complications, thus allowing safe, lifelong, catheter-free bladder drainage [4].

## 3. Indications

There are four major reasons to consider an ileovesicostomy in the modern day.

1. *Neurogenic bladder patients*, who wish to avoid the long-term complications of chronic suprapubic or urethral catheter drainage, yet are unable or unwilling to use CIC or a continent diversion such as a continent catheterizable stoma (e.g., Mitrofanoff or Monti procedure). Ileovesicostomy may be especially indicated in those patients that also need a bladder neck closure, as patient and surgeon prefer the more reliable urinary egress provided by ileovesicostomy over Mitrofanoff, which has nearly a high chance of requiring eventual surgical revision [8].
2. *Cases of intractable lower urinary tract obstruction*, such as bladder neck contracture, in a patient who is unable or unwilling to have an alternative suprapubic diversion (e.g., suprapubic tube or Mitrofanoff).
3. *Lower urinary tract disaster* that may be caused by radiation therapy for prostate cancer (e.g., urethral stricture, and/or urethral-rectal fistula, and/or incontinence, and/or a small capacity bladder).
4. *Urethrocutaneous fistula* into a decubitus ulcer in a spinal cord injury patient [9].

## 4. Performance Characteristics of Ileovesicostomy for Neurogenic Bladder

McGuire and associates [10] reported that ileovesicostomy maintains a detrusor leak point pressure less than 40 cm of water and preserves upper tract function as well or better than the traditional treatments of anticholinergic medications and CIC. However, this patient population was “self-selected” as they generally chose to abandon CIC either by choice or through lack of home care support. In a long-term study, Leng reported that yearly measures of detrusor leak point pressure less than 40 cm of water were achieved in the 38 of 41 of these patients (93%) [11].

In a large series of ileovesicostomy patients with neurogenic bladder, Schwartz et al. [12] reported in 23 patients that the creation of low-pressure urine collection system could eliminate the associated problems with urinary incontinence, that is, infection, calculi, and urethrocutaneous fistulae. The authors asserted that preservation of the native vesicoureteral junction and neurologic urinary function could be attained with an ileovesicostomy, but not with cystectomy and ileal loop. The surgical morbidity associated with cystectomy is avoided, and it is technically easier to perform. The ileovesicostomy has the additional advantage of utilizing the native trigone hence maintaining the native ureter's nonrefluxing mechanism [12].

Rivas and associates described the cutaneous ileocystostomy or “bladder chimney” in the treatment of severe neurogenic vesical dysfunction. Their results indicated that this procedure was a valid alternative for patients with “a bladder capacity less than or equal to 200 mL, recurrent febrile urinary tract infection, and urinary incontinence despite an indwelling urethral catheter.” In the 11 patients studied, the need for long-term urinary catheterization was eliminated with effective low-pressure urinary stomal drainage [13].

## 5. Indications for the Ileovesicostomy for Neurogenic Bladder

In the neurogenic bladder patient who is unable to store urine, Elliot and associates indicated that medical therapy was considered first treatment. Among surgical treatments, the “gold standard” operations included supravesical diversion or augmentation cystoplasty [14]. Ileovesicostomy may be particularly useful in those with poor bladder compliance. It is known that a poorly compliant bladder can develop in about 10% percent of patient with suprasacral spinal cord injuries and 50% with sacral level injuries [4]. With spinal cord injury, a correlation exists with upper tract complications and poor bladder compliance. This has been associated with radiographic upper tract abnormality, vesicoureteral reflux, pyelonephritis, and upper tract stones [4, 15]. Reliable low-pressure egress of urine without the need for catheters provided by the ileovesicostomy is an ideal answer to this problem.

Older series often advocate the wide use of cystectomy and ileal conduit to treat neurogenic bladder. However, cystectomy is unnecessarily morbid and is made largely unnecessary by the ileovesicostomy operation. Also, the lifetime risk of ureteroileal obstruction after cystectomy is higher than most want to admit—a complication that is avoided with ileovesicostomy. Recently, in a cohort of 553 cystectomy patients, Msezane and associates report a ureteral anastomosis stricture rate of 7% [16]. We reserve cystectomy/ileal loop only in those patients who have a completely acontractile bladder (in which an ileovesicostomy is thought to empty poorly by some experts) or in those with other bladder pathology requiring bladder removal (fistula, etc.) [5, 12].

## 6. Outcomes/Complications of Ileovesicostomy

In 1994, Schwartz and associates reported 23 patients who underwent ileovesicostomy. Of these cases, 17 patients were quadriplegic, five had lower spinal cord abnormalities, and one patient had a “watering pot perineum.” They reported a mean followup of 45 months. Twenty-one of 23 (91%) patients had egress of urine at bladder pressure less than 20 cm of water. Early complications included pneumonia, bladder outlet obstruction, poor drainage, and increased leak point pressure. Late complications included stomal stenosis, parastomal hernia, and detrusor hyperreflexia [12].

In a quadriplegic population, Mutchnik and associates reported six consecutive male patients who underwent ileovesicostomy for bladder management. The majority of patients reported being displeased with current bladder regimens that included CIC in two patients, suprapubic catheter in three patients, and urethral catheter in one. Overall, patients did well, but the best outcomes were seen in patients with low (<100 mL) preoperative urine residuals. Complications were relatively uncommon: one patient (17%) had a urinary tract infection, associated with a postoperative residual volume greater than 100 mL, while one (17%) had continued urethral urine leakage and was managed successfully with perineal closure of the urethra.

In 1999, Gudziak et al. [5] reported their experience of 13 patients which had undergone incontinent ileovesicostomy. Preoperatively, their patient population suffered chronic urinary tract infections, incontinence, or urethral erosion. The mean followup was 23 months. Fourteen concomitant procedures were preformed in 11 patients and four complications were identified including stomal stenosis (7%), wound infection (7%), and two patients with ileus (15%) which resolved spontaneously. There was improvement in vesicoureteral reflux, and mean detrusor leak point pressures dropped to an average of 8 cm of water. No associated mortality was seen, and no patients developed renal insufficiency postoperatively [5].

Tan et al. [17] reported their experience of 50 patients who had undergone ileovesicostomy with a mean followup of 26 months. Their patient population primarily consisted of neurogenic bladder cases with 21 patients having spinal cord injuries and 19 patients having multiple sclerosis. Eighty-eight percent had preoperative incontinence despite conservative bladder management, with 37 patients (74%) having indwelling urethral and/or suprapubic catheter drainage devices. Thirty-nine concomitant procedures were performed, usually cystotomy closures. The incidence of urinary tract comorbidities decreased from 3.4 per patient per year to 1.2 per patient per year. Continence was achieved in 36 (72%) patients. Twenty-eight stoma complications were noted in 19 patients: 14 ill-fitting appliance, eight stomal stenosis, three ulcerations, two retractions, and one hernia. Twenty-three mechanical complications were noted in 11 patients primarily related to ileovesicostomy obstruction (12%), stomal stenosis (12%), ileal-kinking secondary to redundancy (10%), mucus plugging (8%), and ileovesicostomy stricture (4%) (Table 1). Interestingly, reoperation of 77 procedures occurred in 27 patients ranging from ileovesicostomy revisions to urethral closure procedures. There was no associated mortality within the first 12 months [17].

Overall, various authors have reported their complications associated with ileovesicostomy. These outcomes range as follows: urethral incontinence rates (16–88%), urinary tract infection (16%), urinary fistula (32%), wound infection (7–34%), hematuria (18%), stomal stenosis (7–16%), ileovesicostomy obstruction (12%), fascial stenosis (12%), and small bowel obstruction/ileus (15%).

## 7. Laparoscopic Ileovesicostomy

As the world of minimally invasive surgery has grown, the attempt to perform ileovesicostomy via the laparoscopic approach is evolving. The proposed benefits for laparoscopic approach include comparable operative time, decrease in blood loss, and decreased postoperative convalescence. Currently, there are two case reports in humans describing laparoscopic ileovesicostomy. The first case was performed in 240 minutes and blood loss of 100 mL. The patient had an uneventful postoperative course and was discharged on hospital day four [18]. The second case study described the first successful pure laparoscopic approach performed in 270 minutes and an estimated blood loss of 50 mL [19].

## 8. Predicting the Need for Bladder Neck Closure

In performing an ileovesicostomy, the question will arise as whether to routinely close the bladder neck in order to fix continued urinary incontinence from the patient's urethra. The literature does not readily identify patients that would benefit from this procedure. Usually we do not perform urethral/bladder neck closure at the time of ileovesicostomy unless we have objective evidence that it is required (such as a patient with a pre-existing suprapubic tube who has persistent total incontinence through the urethra). Only one study addressed this issue in patients with ileovesicostomy for neurogenic bladder; Mutchnik had one patient (17%) who subsequently required bladder neck closure secondary to persistent urethral leakage. Another author reported no patients in their series of 13 patients, who required bladder neck closure [5]. However, patients who are treated for urethral destruction have a high rate of requiring subsequent urethral closure (perhaps because the urethral destruction is associated with concomitant bladder neck destruction). In addition, Schwartz et al. provide some helpful guidance, as they performed bladder neck closure at the time of ileovesicostomy in women patients when fluorourodynamic studies demonstrated poor proximal urethral function.

## 9. Detroit Receiving Hospital Experience

**Figure 1:**
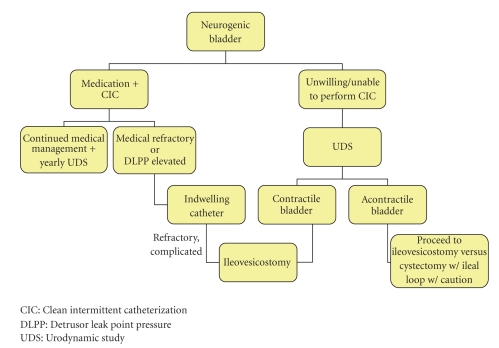
Decision tree for the management of neurogenic bladder.

After approval by the Human Investigations Committee at Wayne State University, we evaluated our patients who had undergone ileovesicostomy to characterize the common complications following the procedure and determine success rates. Patients were included if 18 years of age or older and had undergone an ileovesicostomy for a five-year period ending September 2007 at Detroit Receiving Hospital. Patients were treated according to our algorithm (Figure 1).

Extraction of patient information from the surgeon's procedural logs from the prescribed time period yielded eight patients: 5 men and 3 women. The mean age was 33 years (range 25–37). The mean weight and BMI was 65 ± 22 kg and 25 ± 8 kg/m^2^, respectively. Patients had an average length of stay of 12 days, however some patients had multiple combined general and urological surgical procedures which extended their stay. All patients discharged to home with the exception of one patient who returned to an extended care facility. In this urban population, substance abuse was common: 75% tobacco use, 50% ethanol use, and 50% recreational drugs use. The most common diagnosis for surgical consideration was neurogenic bladder with persistent urine leakage (Table 2).

The primary etiology in our patients was spinal cord injury in seven, five as a result from complications of gun shot wounds. The others were a wrestling injury and a diving injury. One patient was classified as a quadriplegic and six were paraplegic. The eighth patient suffered from total urinary incontinence as a result from cervical cancer and had previously underwent total abdominal hysterectomy and bilateral salpingoophrectomy followed by radiation to the pelvis.

Preoperatively, the majority of our patients were managed with urethral catheters. Other techniques were also employed: CIC, incontinence pads, chronic suprapubic catheterization, and failed enterocystoplasty with Mitrofanoff appendicovesicostomy. Followup was possible in 7 of 8 patients; all were alive and well postprocedure.

A total of six complications were identified in eight patients. Complications associated with our ileovesicostomy patient population included one urinary tract infection (16%), one wound dehiscence (16%), one blood stream infection (16%), and two patients who developed temporary ileus (32%). One patient (16%) with a history of total incontinence secondary to high-dose cervical radiation therapy developed a rectovaginal and vesicourethral fistula after the ileovesicostomy procedure. Although Tan et al. [17] reported significant reoperation rates, our experience demonstrates much lower reoperative and complication rates (Table 2).

## 10. Ileovesicostomy Avoids the Many Potential Complications of Chronic Catheterization

Ileovesicostomy serves as an alternative in those patients with chronic outlet obstruction who have failed conservative management. The expected complication rate from ileovesicostomy is enumerated previously in this paper. The complications of chronic urethral or suprapubic catheterization are well known, but harder to quantify in the literature. Schwartz et al. reported 30% patients with progressive incontinence or urethrocutaneous fistulas, 39% with urosepsis, 39% with recurrent upper and/or lower tract urolithaisis, 13% had autonomic dysreflexia, and 17% had worsening hydronephrosis and/or ureteral reflux [12]. In a related article, Larsen et al. reported 49 of 56 (88%) of urethrally catheterized patients experienced a total of 202 complications including renal damage, UTIs, stones, urethral erosions, strictures, abscesses, and 2 deaths associated to urosepsis [20].

Patients with suprapubic catheters were noted to have complications too. In the preanticholinergic era, patients with suprapubic tubes appeared to suffer renal deterioration equivalent to 20 years of urethral catheterization, but after only 5 years time [21]. Probably, modern use of anticholinergics has improved upper tract protection [22]. Finally, urothelial malignancy is a dreaded complication of prolonged urinary catheterization. In the Department of Veterans Affairs study, chronic catheterization led to bladder cancer in 0.4% of patients [23].

Upper tract deterioration is another complication of chronic catheterization that might be avoided with ileovesicostomy. Ku et al. report an incidence of upper tract deterioration in 52% and 26%, pyelonephritis in 41% and 31%, and renal stones in 21% and 36% of patients with SCI managed with urethral catheter, and suprapubic catheter respectively. This was a long-term study with a mean followup of 25.2 years [24]. There also appears to be a greater incidence of bladder calculus formation in patients with a urethral catheter as compared to suprapubic cystotomy [25].

## 11. Conclusions

Currently, debate exists amongst practitioners regarding neurogenic bladder patients where ileovesicostomy should be placed in the algorithm for treatment. The current practice standard is trial of management with CIC, followed by indwelling urinary catheter (urethral versus suprapubic cystotomy), and later, operative maneuvers to eliminate the chronic catheter altogether. Operative intervention consists of cystectomy with ileal diversion, augmentation cystoplasty with or without appendicovesicostomy, or ileovesicostomy. Multiple authors have suggested that in patients with severe lower urinary tract symptoms and who are unable to perform CIC and/or refractory to medical therapy, ileovesicostomy should be the procedure of choice. In light of the known complications associated with chronic indwelling urinary catheters, we have found this procedure beneficial, as well as easy to perform, generally successful, and durable. Postoperative or intraoperative closure of the incompetent bladder neck might sometimes also be required in the ileovesicostomy patient.

## Figures and Tables

**Table 1:** Comparison of complications.

Author	Year	*N*	Indication	Complications
Schwartz et al. [12]	1994	23	SCI, MS, CP, myelomeningocele	Early: pneumonia, bladder outlet obstruction, increased LPP
				Late: stomal stenosis, parastomal hernia, detrusor hyperreflexia

Mutchnik et al. [6]	1997	6	SCI	UTI, urethral urine leakage, urinary retention

Gudziak et al. [5]	1999	13	SCI, MS, tuberculosis meningitis	Stomal stenosis, wound infection, ileus, vesicovaginal fistula

Leng et al. [11]	1999	38	SCI, infracervical SCI, MS, other	Early: stomal stenosis, dysreflexia, wound infection, stomal prolapse/stenosis, SBO
				Late: renal and bladder calculi, retracting stoma, UTI, fascial stenosis, ileal limb kink, stricture

Tan et al. [17]	2008	50	SCI, MS, spina bifida, radical retropubic prostatectomy	Stomal complications (stenosis, ulcerations, retractions, hernia), obstruction, stomal stenosis, ileal-kinking, mucus plugging, stricture, wound infections, SBO/ileus, wound dehiscence/evisceration, intra-abdominal abscess, hernia

Zimmerman		8	SCI, fistula	UTI, wound dehiscence, blood stream infection, ileus

SCI: spinal cord injury, MS: multiple sclerosis, UTI: urinary tract infection, SBO: small bowel obstruction, CP: cerebral palsy, LPP: leak point pressure.

**Table 2:** Patient characteristics.

Patient no. (Age, years)	Sex	Etiology	Preoperative management (years)	Followup (months)	Concomitant surgery	Reoperative procedure
1 (27)	M	SCI (C6)	Chronic urethral catheter (3)	Unable to contact	None	None

2 (33)	M	SCI (T11)	CIC (14), chronic urethral catheter (1)	45.1	None	None

3 (41)	F	Vesicovaginal fistula, history of cervical CA with hysterectomy and radiation	Incontinence pads	6	Urethral closure	Vaginoscopy, cystogram, vaginogram

4 (34)	M	SCI (T10)	CIC (1)	26.6	None	Reop 1: urinary fistula closure, urethral closure
						Reop 2: enterocystoplasty, Mitrofanoff, bladder neck closure

5 (27)	M	SCI (T10)	SP catheter, CIC (2)	38.9	None	None

6 (37)	F	SCI (T3)	Cystoplasty, Monti catheterizable stoma, cystoscopy with urethral bulking agent injection (24)	Failed to return to clinic	Urethral closure	None

7 (25)	M	SCI (T3)	Chronic urethral catheter (2)	1.7	Gastrocutaneous fistula repair	Urethral bulking agent injection

8 (37)	F	SCI (T4)	SP catheter (12)	36.6	SP tube closure	None

M: male, F: female, SCI: spinal cord injury, C: cervical, T: thoracic, CA: cancer, CIC: clean intermittent catheterization, SP: suprapubic, Reop: Reoperation.

## Abbreviations

BMI: Body mass index
CIC: Clean intermittent catheterization
DLPP: Detrusor leak point pressure
SCI: Spinal cord injury
UTI: Urinary tract infection.
